# Mixtures of prion substrains in natural scrapie cases revealed by ovinised murine models

**DOI:** 10.1038/s41598-020-61977-1

**Published:** 2020-03-19

**Authors:** Tomás Barrio, Hicham Filali, Alicia Otero, Jessica Sheleby-Elías, Belén Marín, Enric Vidal, Vincent Béringue, Juan María Torres, Martin Groschup, Olivier Andréoletti, Juan José Badiola, Rosa Bolea

**Affiliations:** 1 Centro de Encefalopatías y Enfermedades Transmisibles Emergentes, Facultad de Veterinaria, Instituto Agroalimentario de Aragón - IA2 (Universidad de Zaragoza - CITA), 50013 Zaragoza, Spain; 2 Priocat Laboratory, Centre de Recerca en Sanitat Animal (CReSA), UAB-IRTA, Universitat Autònoma de Barcelona (UAB), 08193 Bellaterra, Barcelona Spain; 3 UMR Virologie Immunologie Moléculaires (VIM-UR892), INRA, Université Paris-Saclay, 78352 Jouy-en-Josas, France; 4 Centro de Investigación en Sanidad Animal, CISA-INIA, 28130 Valdeolmos, Madrid Spain; 5 Institute of Novel and Emerging Infectious Diseases, Friedrich-Loeffler-Institute, Südufer 10, 17493 Greifswald-Isle of Riems, Germany; 6 UMR INRA ENVT 1225- IHAP, École Nationale Vétérinaire de Toulouse, 31076 Toulouse, France

**Keywords:** Neurodegeneration, Prion diseases

## Abstract

Phenotypic variability in prion diseases, such as scrapie, is associated to the existence of prion strains, which are different pathogenic prion protein (PrP^Sc^) conformations with distinct pathobiological properties. To faithfully study scrapie strain variability in natural sheep isolates, transgenic mice expressing sheep cellular prion protein (PrP^C^) are used. In this study, we used two of such models to bioassay 20 scrapie isolates from the Spain-France-Andorra transboundary territory. Animals were intracerebrally inoculated and survival periods, proteinase K-resistant PrP (PrP^res^) banding patterns, lesion profiles and PrP^Sc^ distribution were studied. Inocula showed a remarkable homogeneity on banding patterns, all of them but one showing 19-kDa PrP^res^. However, a number of isolates caused accumulation of 21-kDa PrP^res^ in TgShp XI. A different subgroup of isolates caused long survival periods and presence of 21-kDa PrP^res^ in Tg338 mice. It seemed that one major 19-kDa prion isoform and two distinct 21-kDa variants coexisted in source inocula, and that they could be separated by bioassay in each transgenic model. The reason why each model favours a specific component of the mixture is unknown, although PrP^C^ expression level may play a role. Our results indicate that coinfection with more than one substrain is more frequent than infection with a single component.

## Introduction

Transmissible spongiform encephalopathies (TSEs), also known as prion diseases, are a group of rare, fatal and progressive neurodegenerative disorders that affect both animals and human beings. TSEs are caused by non-conventional etiologic agents called prions. According to the widely accepted prion hypothesis^1^, prions consist exclusively of a pathogenic protein conformer, termed PrP^Sc^, that derives from the physiological, host-encoded cellular prion protein (PrP^C^)^1–3^ via a post-translational conformational change that is triggered by PrP^Sc^ itself in a feed-back manner^4^. PrP^Sc^ accumulates in nervous tissue^5^ and through a yet unclarified mechanism gives rise to neuronal death, neuron loss and vacuolization^6,7^, together with astrogliosis, microgliosis^8–10^ and other neuroinflammatory phenomena^11^. This progressive degeneration of the central nervous system manifests as a set of neurological signs appearing after long incubation periods.

Although, according to the protein-only model^1^, the agent responsible for these disorders lacks nucleic acids, the existence of several phenotypic variants has been proved for different TSEs. This was first described for scrapie when two different clinical syndromes were observed in scrapie-infected goats, which were reproducible by intracerebral inoculation^12^. Prion variants associated to these different phenotypes were termed “strains”, by analogy with other infectious agents. Further demonstration of the existence of scrapie strains was addressed by studies in wild type mice^13,14^, which also established a methodology to discriminate them according to survival periods and lesion profiles^15–17^.

Other typing approaches based on the assessment of PrP^Sc^ distribution^18^ or the molecular characterization of the prion protein have been successfully employed to discriminate between BSE and scrapie^19^ and among scrapie strains^18,20,21^.

The existence of prion strains can be accommodated within the protein-only hypothesis through the notion that the abnormal conformation of PrP^Sc^ is not unique. In this model, prion strains are encoded in the conformation of PrP^Sc^ molecules, which can adopt several folding states that are associated to different pathological features^22–24^. Within this framework, the conformational selection model^25^ proposes that a given amino acid sequence for PrP^C^ allows a limited portfolio of conformations, and thus only a number of prion strains will induce its misfolding while others will not be able to template its conversion into a disease-associated conformation. As a consequence, the degree of overlapping between the *Prnp* gene sequence of host and donor influences the capability of an isolate to transmit the disease, which provides a molecular explanation for the transmission barrier phenomenon.

The use of wild-type mouse lines expressing different alleles of the murine *Prnp* gene allowed the differentiation of at least 20 scrapie strains^14^. However, wild type mice-based typing methodologies may not be reliable since many scrapie isolates cannot transmit to these models, including isolates of classical scrapie^26,27^, unconventional scrapie isolates such as CH1641^28^ and atypical scrapie^29,30^. When transmission to wild-type mice is achieved, survival periods tend to be very prolonged and highly variable^26^. This phenomenon is known as transmission barrier^31,32^ and was also observed in experimental transmissions of other TSEs to rodent models^33^. Differences in PrP^C^ amino acid sequence between donor and host, both on interspecies and intra-species transmission experiments, are frequently acknowledged as the main molecular determinant of transmission barriers, and are also responsible for subclinical infections^34^. Moreover, according to the conformational selection model^25^, heterotypic interaction between PrP^Sc^ and PrP^C^ can favour the propagation of PrP^Sc^ conformers present in small quantities^25,33^, sometimes referred to as “substrains”, and also components or “quasi-species”^35^. Consequently, studying scrapie strain variability by means of bioassay in a model expressing a non-ovine PrP^C^ may alter the original portfolio of prion variants to the point that it keeps little if any resemblance with the original sheep scrapie strain range.

In this line, the use of homologous murine models, i.e. mice expressing ovine PrP^C^ on a murine *Prnp*^*-/-*^ background, may be crucial to faithfully recapitulate the actual variability of scrapie prion strains present in sheep populations. To this end, several distinct transgenic mouse lines, carrying different alleles (A^136^R^154^Q^171^, V^136^R^154^Q^171^) of the ovine *Prnp* gene, have been created^36^. These ovinised models show enhanced susceptibility to direct sheep scrapie infection^37^ and have proven useful in studies seeking to characterize field sheep scrapie isolates^20,21,26,27,38–40^. In this study, we used two of such models, the TgShp XI line (expressing ovine ARQ PrP^C^)^41^ and the Tg338 line (expressing ovine VRQ PrP^C^)^42^ to study the variability of prion strains and substrains present in the tissues of ten naturally scrapie-infected sheep coming from different outbreaks of the disease within the Spain-France-Andorra transboundary territory.

Scrapie is present as an enzootic disease in small ruminant populations in most European countries, with a prevalence of 7.84 cases per 1000 tested animals in Spain (year 2017)^43^ and 3.92 cases per 1000 tested animals in France (2002–2007)^44,45^. Therefore, its economic and sanitary impact is meaningful. Characterizing and holding control of the variety of enzootic scrapie strains present in the small ruminant population through the Spain-France-Andorra transboundary territories is crucial for scrapie control and eradication purposes and for public health.

## Results

### Biochemical characterization of nervous and lymphoid tissue sheep scrapie field isolates discloses different biochemical patterns

Ten naturally scrapie-infected sheep were used for this study; six of them were at the terminal stage of the disease while four were in a preclinical / early clinical phase. With the aim of including as many different scrapie strains as possible, the selection was done according to two criteria: (a) animals coming from geographically distant scrapie outbreaks (Supplementary Fig. S1), and (b) animals presenting distinct clinical signs. Clinical stage, clinical signs, genotype, date of sacrifice, age at sacrifice and flock of origin of sheep are indicated in Table 1. Both nervous (medulla oblongata) and lymphoid tissues (mesenteric lymph node) of sheep were subjected to biochemical analyses prior to bioassay.

**Table 1:** Clinical stage, clinical signs, genotype, date and age at sacrifice and flock of origin of sheep included in the study, and references of the inocula prepared from their tissues.

ID	Clinical stage	Clinical signs	Genotype	Date of sacrifice (d/m/y)	Age at sacrifice (years)	Flock of origin	Inocula
**Sheep #1**	Terminal	Cachexia, muscle wasting	ARQ/ARQ	25/01/2007	5.5	Zaragoza-1	1 N
							1 L
**Sheep #2**	Terminal	Cachexia, ataxia, alopecia	ARQ/ARQ	03/11/2006	6.5	Zaragoza-2	2 N
							2 L
**Sheep #3**	Terminal	Cachexia, ataxia, hyperexcitation	ARQ/ARQ	18/08/2008	5	Zaragoza-3	3 N
							3 L
**Sheep #4**	Terminal	Cachexia, ataxia, alopecia	ARQ/ARQ	21/02/2008	4	Huesca-1	4 N
							4 L
**Sheep #5**	Terminal	Apathy, ataxia, teeth grinding	ARQ/ARQ	19/12/2007	5.6	Teruel-1	5 N
							5 L
**Sheep #6**	Terminal	Cachexia, ataxia	ARQ/ARQ	11/06/2008	7	Teruel-2	6 N
							6 L
**Sheep #7**	Early clinical	Poor general condition	ARQ/ARQ	04/12/2008	3.5	Zaragoza-4	7 N
							7 L
**Sheep #8**	Preclinical	No signs	ARQ/ARQ	11/11/2008	1.5	Zaragoza-4	–
							8 L
**Sheep #9**	Early clinical	Local alopecia	ARQ/ARQ	04/12/2008	4.5	Huesca-2	9 N
							9 L
**Sheep #10**	Preclinical	No signs	ARQ/ARQ	11/11/2008	1	Zaragoza-4	–
							10 L

**Figure 1:**
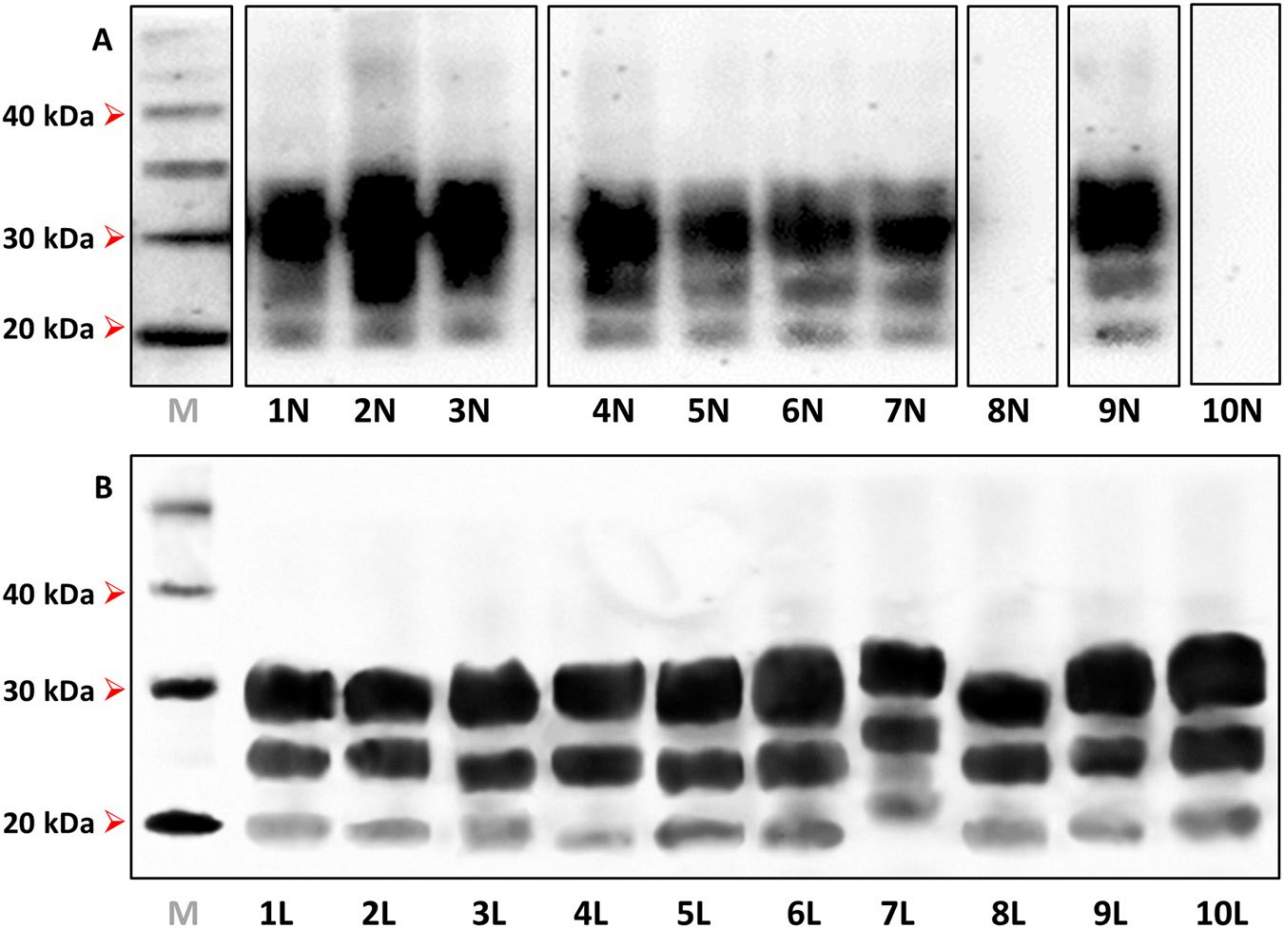
Western blot of inocula sourced from sheep tissues. (**A**) Inocula from medulla oblongata; (**B**) inocula from mesenteric lymph node. Note that all but one samples had banding patterns with the non-glycosylated (NG) band at 19 kDa. *M*: molecular weight marker; kDa: kilodaltons. Notice that cropped images are presented here; for the full image, see Supplementary Figure S3.

Medulla oblongata of sheep #1 to #7 and sheep #9 were positive on Western blot for PrP^res^ and showed a banding pattern with the non-glycosylated (NG) bands at around 19 kDa and predominance of diglycosylated (DG) species (Fig. 1A). These features resemble those of the experimental isolate CH1641^46^, natural CH1641-like isolates from field cases^47,48^ and other natural sources found in the Spain-France-Andorra transboundary territories^49^.

In contrast, medulla oblongata of sheep #8 and #10 were negative on Western blot (Fig. 1A). Since this is likely to be associated with reduced infective titres, they were not used in bioassay.

The presence of PrP^res^ in the lymphoreticular system was demonstrated by Western blot of mesenteric lymph nodes from all sheep (Fig. 1B). All but one showed banding patterns with the NG band at around 19 kDa. In contrast, inocula 7 L showed a different biochemical profile characterized by a NG band with higher weight (~21 kDa), suggesting the presence of a different PrP^Sc^ conformer. 21-kDa PrP^res^ banding patterns have been commonly observed in the most widely used types of experimental scrapie, including SSBP/1 and Dawson isolates^33,46,50,51^, and in several natural scrapie cases^49^.

The presence of different biochemical signatures among our isolates suggests some degree of variability in the prion agent they bear. However, to accurately assess the actual range of prion variants present in our inocula, the disease phenotype in mice needs to be analysed. For that, sheep tissues were used to prepare nervous (N) and lymphoid (L) tissue-derived inocula. Two passages of these inocula were performed in each of the following transgenic lines: TgShp XI mice, which express ovine ARQ PrP^C^ with expression levels of between 4 and 8-fold compared to that of sheep brain^41^, and Tg338 mice, which express ovine VRQ PrP^C^ with expression levels of approximately 8-fold those of sheep brain^37^.

### Bioassay in sheep PrP^C^-expressing transgenic mice suggest the presence of more than one prion variant

Survival periods and attack rates of first and second-passage TgShp XI and Tg338 mice and banding patterns of second-passage spinal cord pools are presented in Table 2. Banding patterns on first passage were coherent with those of second passage.

**Table 2:** Banding patterns of source sheep tissues and survival periods, attack rates and banding patterns of experimentally challenged first and second-passage TgShp XI and Tg338 mice.

Inoculum	Sheep	TgShp XI					Tg338				
		1^st^ passage		2^nd^ passage			1^st^ passage		2^nd^ passage		
	Banding pattern	Survival period	Attack rate	Survival period	Attack rate	Banding pattern	Survival period	Attack rate	Survival period	Attack rate	Banding pattern
1 N	19 kDa	196 ± 12	5/5	239 + 56	5/5	19 kDa	150 ± 7	6/6	185 ± 27	6/6	19 kDa
2 N	19 kDa	213 ± 16	6/6	226 + 68	6/6	19 kDa	179 ± 41	5/5	175 ± 29	6/6	19 kDa
3 N	19 kDa	305 ± 62	5/5	312 + 78	5/5	**21 kDa**	313 ± 110	4/5	199 ± 36	5/5	19 kDa
4 N	19 kDa	273 ± 57	6/6	249 + 50	3/3	**21 kDa**	327 ± 163	6/6	195 ± 30	6/6	19 kDa
5 N	19 kDa	235 ± 13	6/6	240 + 44	5/5	**21 kDa**	324 ± 48	5/5	163 ± 5	6/6	19 kDa
6 N	19 kDa	256 ± 57	6/6	202 + 14	5/5	19 kDa	222 ± 82	6/6	200 ± 58	5/5	19 kDa
7 N	19 kDa	346 ± 5	5/5	190 ± 47	4/4	19 kDa	590 ± 58	6/6	189 ± 36	6/6	19 kDa
9 N	19 kDa	381 ± 35	4/4	183 ± 13	6/6	19 kDa	551 ± 48	6/6	188 ± 37	6/6	19 kDa
1 L	19 kDa	455 ± 30	6/6	273 ± 118	12/12	19 kDa	352 ± 22	6/6	231 ± 47	6/6	19 kDa
2 L	19 kDa	454 ± 140	4/4	207 ± 96	5/5	19 kDa	**419** ± **45**	**6/6**	**646** ± **35**	**5/6**	**21 kDa**
3 L	19 kDa	425 ± 83	4/4	330 ± 0	4/4	19 kDa	491 ± 69	4/5	249 ± 26	5/5	19 kDa
4 L	19 kDa	443 ± 22	3/3	203 ± 65	6/6	19 kDa	**503** ± **77**	**6/6**	**490** ± **110**	**3/6**	–
5 L	19 kDa	314 ± 36	5/5	171 ± 20	6/6	19 kDa	**528** ± **29**	**6/6**	**481** ± **159**	**3/5**	–
6 L	19 kDa	489 ± 80	5/5	276 ± 82	6/6	19 kDa	**644** ± **141**	**5/5**	**637** ± **4**	**7/7**	**21 kDa**
7 L	**21 kDa**	487 ± 38	4/4	241 ± 54	5/5	**21 kDa**	431 ± 44	4/4	195 ± 31	6/6	19 kDa
8 L	19 kDa	422 ± 90	6/6	175 ± 58	4/4	19 kDa	**579** ± **118**	**4/4**	**600** ± **40**	**5/5**	**21 kDa**
9 L	19 kDa	510 ± 63	4/4	163 ± 64	3/3	19 kDa	436 ± 103	6/6	201 ± 46	6/6	19 kDa
10 L	19 kDa	451 ± 78	5/5	321 ± 23	3/3	19 kDa	487 ± 81	4/4	163 ± 7	6/6	19 kDa

Survival period provided as mean ± standard deviation (SD).

**Figure 2:**
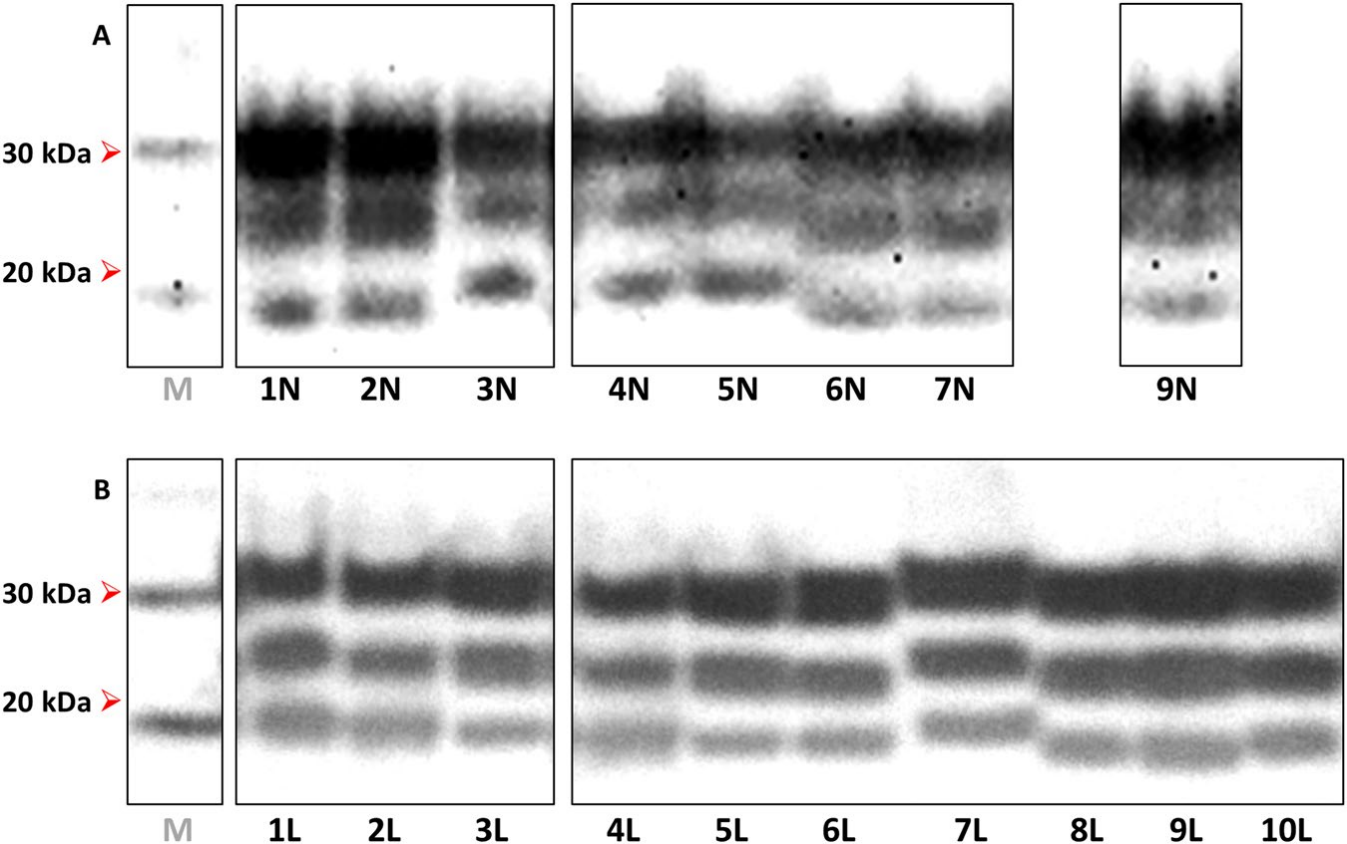
Western blot of spinal cord homogenates from second-passage TgShp XI mice experimentally challenged with (**A**) sheep brain-derived isolates and (**B**) sheep lymph node-derived isolates. Note that most samples had banding patterns with the non-glycosylated (NG) band at 19 kDa, while those of inocula 3 N, 4 N, 5 N and 7 L were at 21 kDa. *M*: molecular weight marker; kDa: kilodaltons. Notice that cropped images are presented here; for the full image, see Supplementary Figure S3.

**Figure 3:**
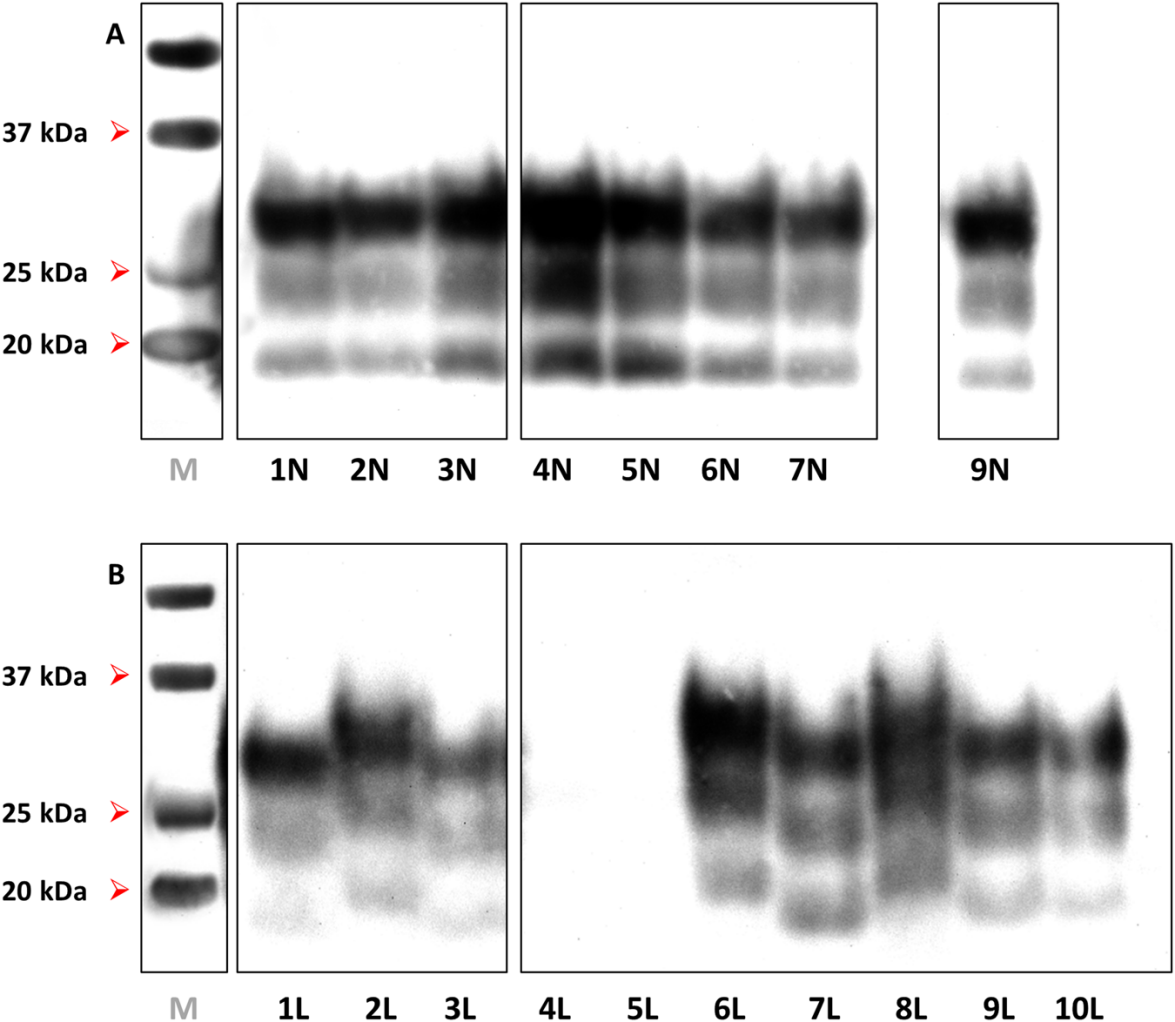
Western blot of spinal cord homogenates from second-passage Tg338 mice experimentally challenged with (**A**) sheep brain-derived isolates and (**B**) sheep lymph node-derived isolates. Note that 13/18 samples were clearly positive with non-glycosylated bands at 19 kDa. 5/18 samples differed from this, either being negative (4 L and 5 L) or having NG bands at 21 kDa (2 L, 6 L and 8 L). *M*: molecular weight marker; kDa: kilodaltons. Notice that cropped images are presented here; for the full image, see Supplementary Figure S3.

A majority of brain-derived inocula (1 N, 2 N, 6 N, 7 N and 9 N) produced clinical disease with accumulation of 19-kDa PrP^res^ in spinal cord of both TgShp XI and Tg338 mice **(**Figs. 2A and 3A). Survival periods ranged from 196 to 381 dpi on first passage and from 183 to 239 dpi on second passage TgShp XI mice. Tg338 mice succumbed to disease showing survival periods between 150 and 590 dpi in first passage and 175–200 dpi on second passage. Preclinical and early clinical sheep-sourced inocula caused longer survival periods than those sourced from terminal sheep on first passage; however, this difference was reduced on second passage.

The rest of brain-derived inocula (3 N, 4 N and 5 N) behaved similarly to the former in Tg338, which showed 19-kDa banding patterns (Fig. 3A) and survival times between 313 and 327 dpi that shortened to 163–199 dpi on second passage, suggesting the propagation of the same prion variant. In TgShp XI, these isolates caused similar or subtly longer survival periods in first (235–305 dpi) and second passage (240–312 dpi). However, the biochemical profile in these mice was characterized by an unglycosylated band at 21-kDa (Fig. 2A). This suggests the presence of minor quantities of a 21-kDa prion conformer, undetectable in the original isolates, that is preferentially amplified by the TgShp XI line.

Regarding lymph node-derived inocula, all but one had a single pattern of transmission to TgShp XI mice characterized by long survival periods on first passage (314–510 dpi) that shortened to 163–330 dpi on second passage, and accumulation of 19-kDa PrP^res^ in their spinal cord (Fig. 2B). In contrast, inoculum 7 L transmitted with similar survival periods (241 dpi on second passage) but, alternatively, triggered 21-kDa PrP^res^ deposition in mice (Fig. 2B), mirroring that of the original isolate (Fig. 1B). Long survival periods on first passage reflect low infectivity titres in lymph node-derived isolates, while on second passage remarkable reductions were observed.

Five of these inocula (1 L, 3 L, 7 L, 9 L and 10 L) transmitted to Tg338 with second-passage survival periods ranging from 163 to 249 dpi. Presence of 19-kDa PrP^res^ was noted in spinal cords of both passages (Fig. 3B), even in the case of inoculum 7 L. The characteristics of these isolates in Tg338 agreed with those of brain-sourced isolates, suggesting that they contain the same type of agent.

In contrast, the other five isolates (2 L, 4 L, 5 L, 6 L and 8 L) transmitted to Tg338 showing unusual transmission characteristics. Longer survival periods were observed in first passage (419–644 dpi), which experienced no reduction or even considerable lengthening on second passage (481–646 dpi). In a number of occasions, even the attack rate was reduced in the second passage, suggesting limited accumulation of infectivity in the spinal cord of first-passage animals. Supporting this notion, Tg338 mice from both first and second passages accumulated reduced quantities of PrP^res^ in spinal cord as demonstrated by the negative results on Western blot of spinal cord homogenates from animals infected with inocula 4 L and 5 L (Fig. 3B). Surprisingly, in the rest of cases (inocula 2 L, 6 L and 8 L), animals accumulated a PrP^res^ with a 21-kDa banding pattern (Fig. 3B), which provides further evidence for the propagation of a different conformer in this mice.

According to transmission patterns and biochemical signatures of the accumulated PrP^res^, the presence of three PrP^Sc^ variants can be envisioned: (i) a 19-kDa variant, present as the major component of the majority of sheep-sourced inocula, which we termed “19K”; (ii) a 21-kDa conformer that appears as the major isoform in inoculum 7 L and as a minor component in other three isolates, and that causes disease in TgShp XI with transmission patterns similar to the 19-kDa variant, hereafter termed “21K-TgShp XI”; and (iii) a 21-kDa conformer, different from the former, that seems to block the propagation of the 19-kDa major component exclusively in Tg338 mice, triggering reduced PrP^Sc^ accumulation in spinal cord and protracting clinical disease, and termed “21K-Tg338”.

### Mice brains show distinct histopathological hallmarks

The characteristics of spongiform change in both transgenic models were fairly similar. Medulla oblongata, ventral mesencephalon and *zona incerta* and ventrolateral nuclei of diencephalon showed invariably the most severe spongiosis. In sharp contrast, vacuoles in cerebellar cortex were usually absent or very scarce. Cortex of superior colliculus and central regions of thalamus suffered a milder vacuolization. In hypothalamus, striatum and septal nuclei, the degree of spongiosis was highly variable among infected mice groups. Finally, frontal and temporo-parietal cortices and hippocampal formation were poorly affected.

Lesion profiles were drawn for each inoculum and murine line. In most cases, curves fitted to a general profile characterized by high scores at brainstem and subcortical structures, moderate scores in cerebral cortices and hippocampus, and low scores in cerebellar cortex. However, a number of isolates triggered lesion profiles with significantly lower vacuolization severity in all areas.

**Figure 4:**
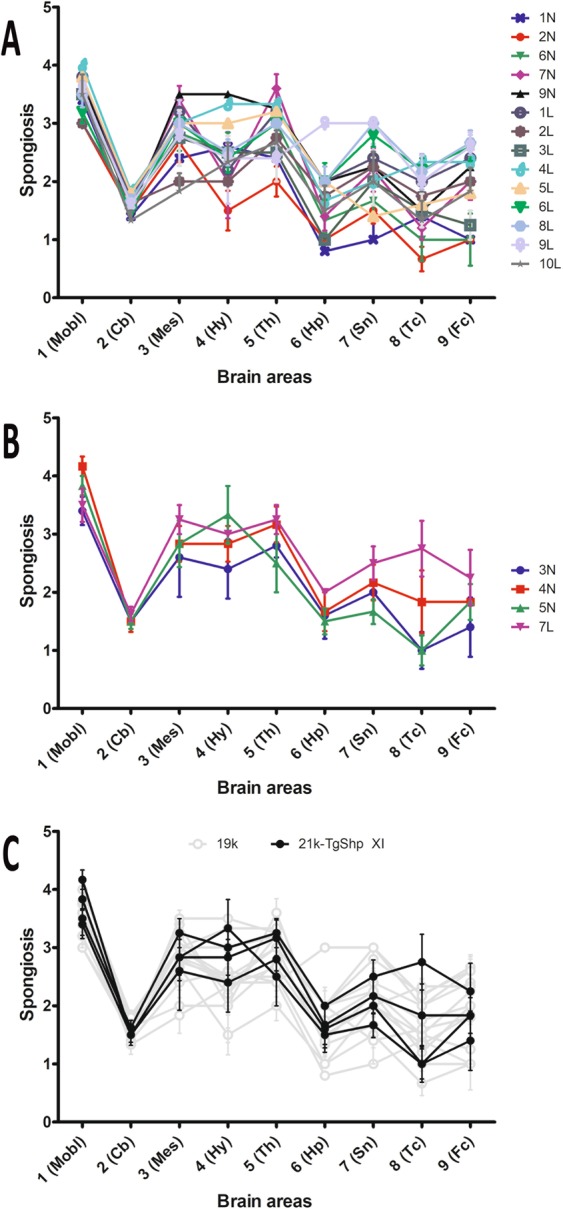
Lesion profiles of second-passage TgShp XI mice. Both inocula associated to the 19K phenotype (**A**) and inocula associated to the 21K-TgShp XI phenotype (**B**) provoked similarly shaped lesion profiles in TgShp XI mice (**C**), most of them characterized by peak scores at mesencephalon and thalamus. The brains of all mice dying after the onset of clinical signs in each challenged group were analyzed to plot lesions profiles (usually 6 and never less than 3 animals per group). Mobl: medulla oblongata, Cb: cerebellar cortex, Mes: mesencephalon, Hy: hypothalamus, Th: thalamus, Hp: hippocampus, Sn: septal nuclei, Tc: cortex at the level of thalamus, Fc: frontal cortex. Error bars represent SEM.

Both 19K substrain (Fig. 4A) and 21K-TgShp XI substrain-containing inocula (Fig. 4B) caused in TgShp XI mice similarly shaped lesion profiles (Fig. 4C), most of them characterized by peaks at medulla oblongata, mesencephalon and thalamus.

**Figure 5:**
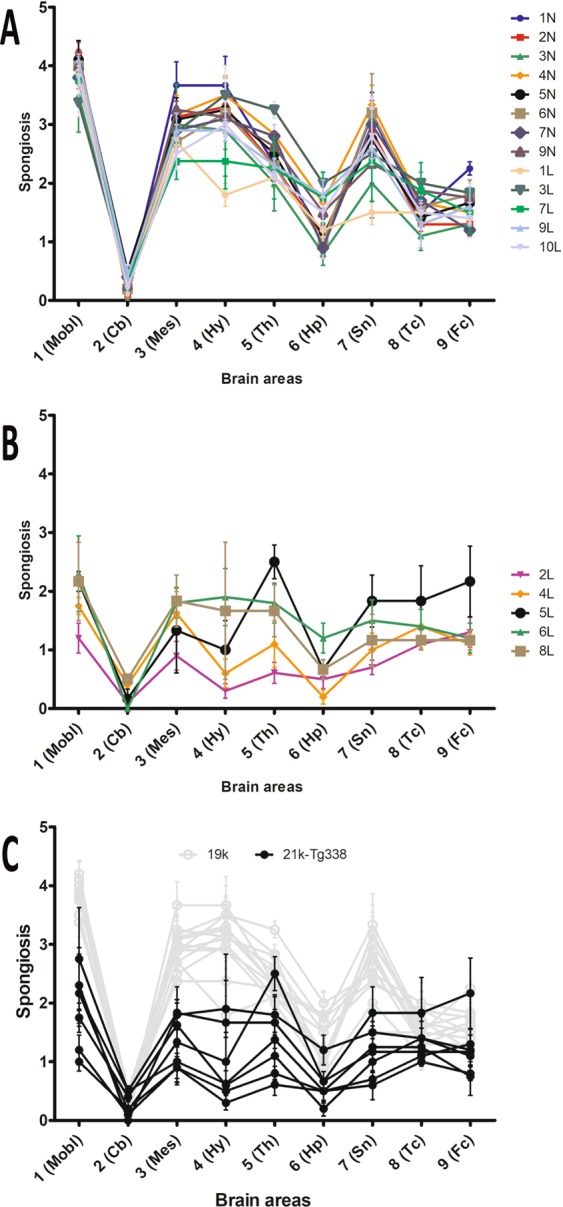
Lesion profiles of second-passage Tg338 mice. Inocula associated to the 19K phenotype provoked similar lesion profiles in Tg338 mice, mostly characterized by peaks at medulla oblongata and thalamus (**A**). However, isolates linked to the 21K-Tg338 phenotype triggered lower and flatter profiles (**B,C**). The brains of all mice dying after the onset of clinical signs in each challenged group were analyzed to plot lesions profiles (usually 6 and never less than 3 animals per group). Mobl: medulla oblongata, Cb: cerebellar cortex, Mes: mesencephalon, Hy: hypothalamus, Th: thalamus, Hp: hippocampus, Sn: septal nuclei, Tc: cortex at the level of thalamus, Fc: frontal cortex. Error bars represent SEM.

In contrast, inocula bearing the 19K substrain (Fig. 5A) and the 21K-Tg338 substrain (Fig. 5B) showed differences in the lesion profiles they triggered in Tg338 mice. The later were characterized by flatter curves, with less evident peaks, and that, on average, were lower than those caused by the other group of isolates (Fig. 5C). This finding reinforces the idea that these inocula contain a different type of prion.

**Figure 6:**
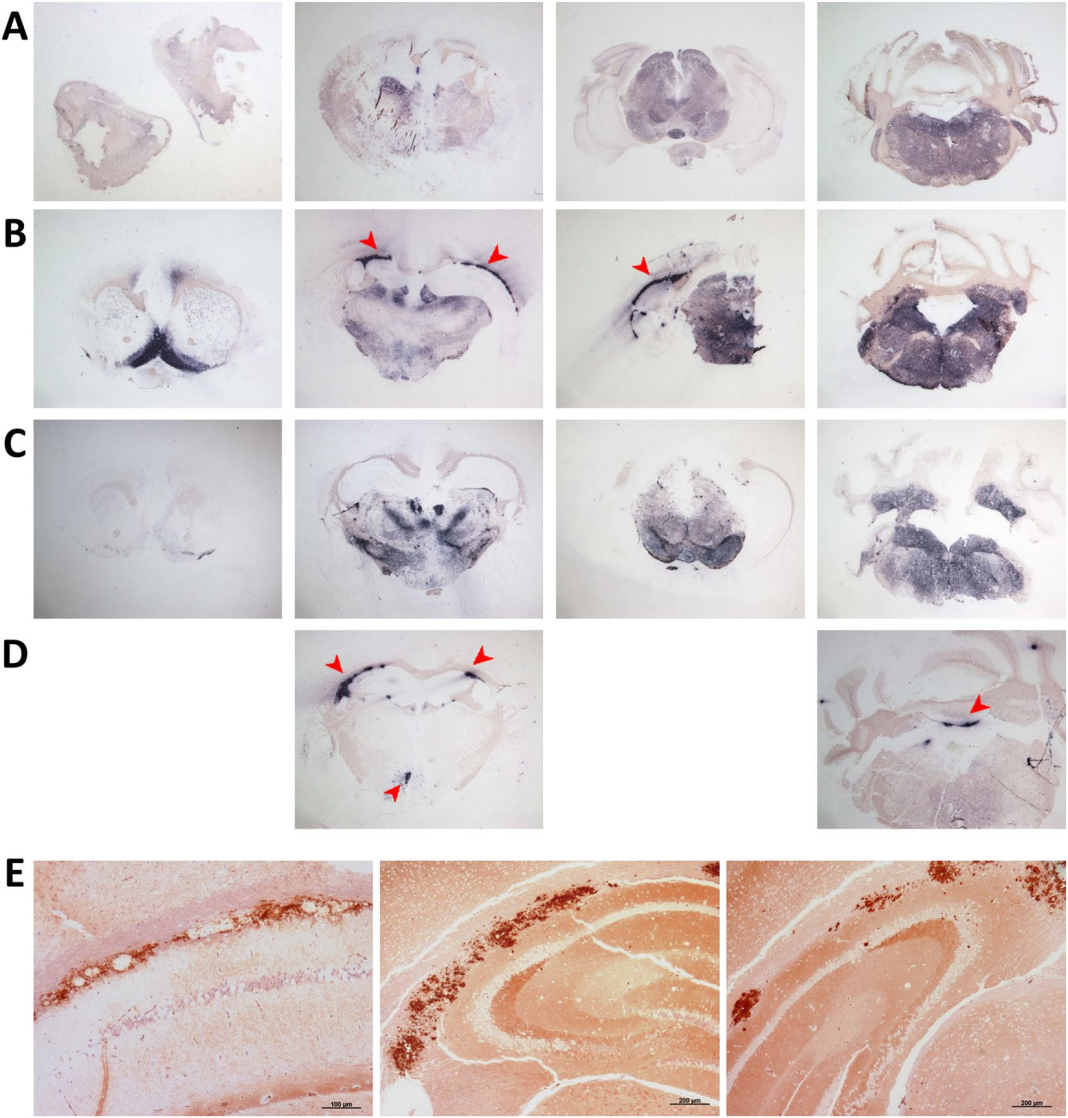
PrP^Sc^ distribution in representative brain sections from TgShp XI mice infected with inocula 2 N (**A**), 3 N (**B**), 7 L (**C**) and 8 L (**D**). Two different trends of PrP^Sc^ accumulation were observed on PET-blots: a generalized low-intensity immunostaining (**A**,**B**,**C**), and a localized high-intensity pattern, usually associated with the alveus of hippocampus (**B**, **D**, arrowheads), and subependymal areas (**D**, arrowheads), and which correlated with coarse/coalescing plaque-like deposits on immunostochemistry (**E**).

PrP^Sc^ distribution was assessed using PET-blot. TgShp XI mice showed in general lower staining intensity in comparison with Tg338 mice. Two trends of PrP^Sc^ deposition could be differentiated. The first was a generalized, widespread low-intensity immunostaining (Fig. 6A,B,C), which correlated with synaptic deposits in IHC and was more intense in medulla oblongata, deep cerebellar nuclei, mesencephalon, medial and lateral nuclei and *zona incerta* of thalamus, hypothalamus and septal area. The second pattern consisted of localized high-intensity, plaque-like deposits, which were found frequently associated to the alveus of hippocampus (Fig. 6B,D) and subependymal areas at the level of 3^rd^ and 4^th^ ventricles (Fig. 6D), and correlated with coarse particulate/coalescing deposits in IHC (Fig. 6E).

**Figure 7:**
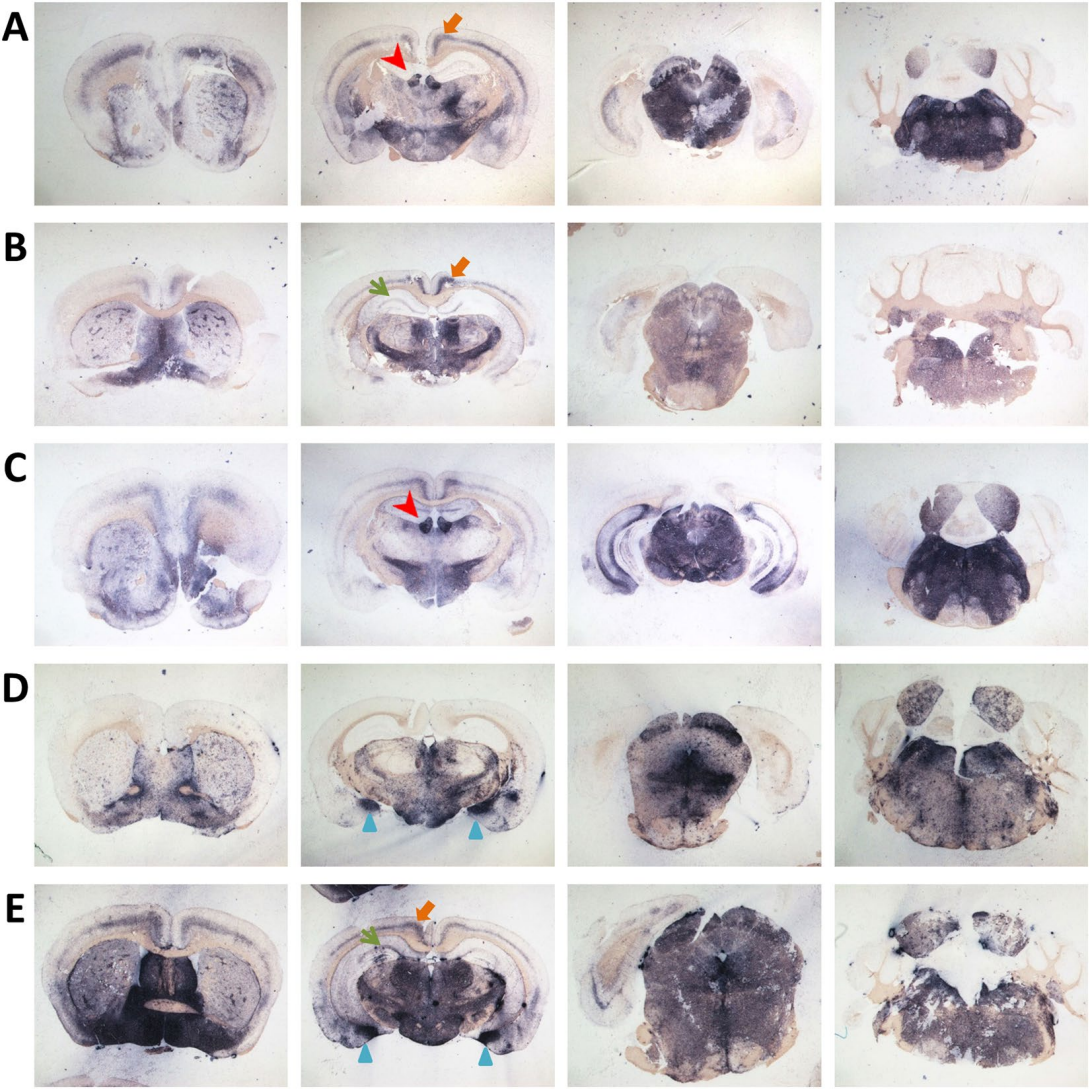
PrP^Sc^ distribution in representative brain sections from Tg338 mice infected with inocula 1 L (**A**), 3 L (**B**), 7 L (**C**) and 8 L (**D**,**E**). A general pattern arose in all examined brain samples consisting of intense staining of brainstem and subcortical areas. Additionally, conspicuous immunostaining was observed in specific structures, including the cingulate gyrus (**A**,**B**,**E**; thick orange arrows), habenular nuclei (**A**,**C**; red arrowheads), and the *stratum lacunosum-moleculare* and the alveus of hippocampus (**B**,**E**; thin green arrows). High-intensity staining of the medial amygdaloid nuclei (**D**,**E**; blue triangles) was observed in a subset of samples obtained from animals challenged with isolates causing prolonged survival periods and low vacuolization scores.

PrP^Sc^ deposits in the brains of Tg338 mice were mostly of the generalized or synaptic type. The distribution pattern was characterized by high staining intensities of brainstem and subcortical areas. Medulla oblongata and deep cerebellar nuclei, mesencephalon, medial and dorsal nuclei of thalamus and *zona incerta*, and hypothalamus were the most affected zones (Fig. 7). A correlation between the distribution of spongiform lesions and PrP^Sc^ deposits was observed in both TgShp XI and Tg338 mice, as seen by the overlapping of the curves and by the positive and statistically significant Spearman’s correlation coefficients (Supplementary Fig. S2).

In addition, mild to intense staining was observed in specific structures, including the cingulate gyrus (Fig. 7A,B,E), the habenular nuclei (Fig. 7A,C), and the *stratum lacunosum-moleculare* and the alveus of hippocampus (Fig. 7B,E). Finally, intense immunostaining of medial amygdaloid nuclei (Fig. 7D,E) was observed exclusively in Tg338 inoculated with isolates 6 L and 8 L, which were associated to long survival periods and low average spongiosis.

**Figure 8:**
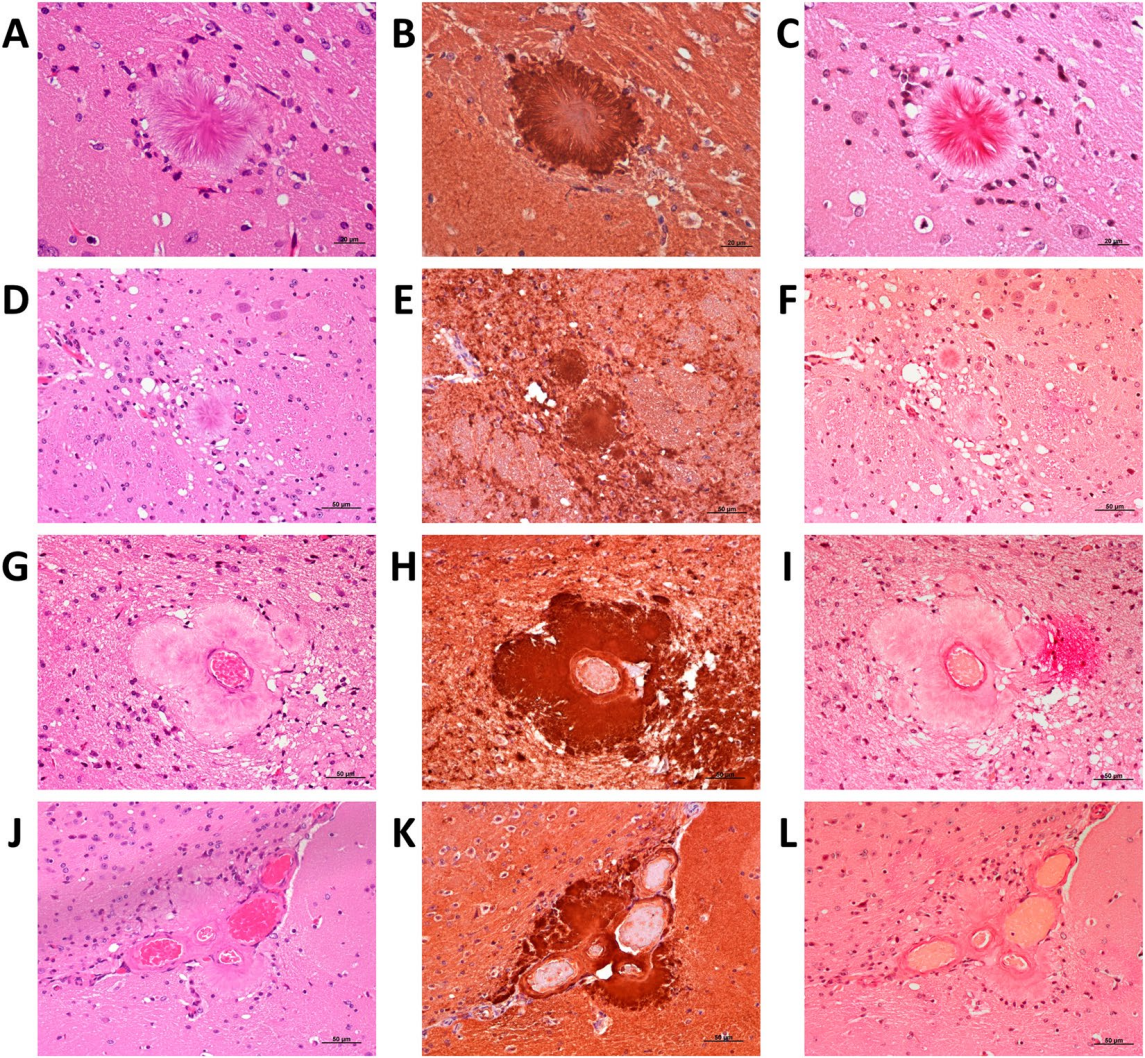
Unicentric (**A** to **C**), florid (**D** to **F**) and amorphous perivascular plaques (**G** to **L**) in brain of second-passage Tg338 mice infected with inocula 6 L and 8 L. Haematoxilin-eosin staining (**A**,**D**,**G**,**J**), immunohistochemistry with anti-PrP mAb SAF84 (**B**,**E**,**H**,**K**) and Congo red staining that proves the amyloid nature of the plaques (**C**,**F**,**I**,**L**).

These same inocula (6 L and 8 L) led to the formation of amyloid plaques in the brain of second-passage Tg338 mice. These plaques were visible on PET-blot as granular plaque-like depositions at the level of thalamus and mesencephalon (Fig. 7D,E), and in haematoxylin and eosin (H&E)-stained slides (Fig. 8A,D,G,J). They were congophilic, which demonstrate their amyloid nature (Fig. 8C,F,I,L), and positive to immunohistochemistry with anti-PrP antibody SAF84 (Fig. 8B,E,H,K). Plaques were usually multicentric or amorphous and were found mostly in a perivascular location and lying beneath the pia mater of frontal, parietal and piriform cortices and cortex of the superior colliculus. In a number of animals, unicentric and occasionally florid plaques were found in the deep layers of medulla oblongata, mesencephalon, hypothalamus and thalamus. Interestingly, this phenotypic feature was not observed in TgShp XI mice.

## Discussion

There is a wide basis of evidence that the characterization of scrapie strains based on data recorded directly from the ovine host is a difficult task^52,53^. Differences in clinical signs of sheep or in lesion profiles and distribution of PrP^Sc^ deposits in their brains may not reflect the presence of different strains, since several uncontrolled factors are known to contribute to this heterogeneity, including age of infection, breed, intercurrent diseases and effects of currently unidentified genes. This problem has been routinely circumvented by bioassaying the isolates in genetically stable rodent models. The advent of transgenic murine lines expressing homologous PrP^C^ has allowed shedding light on the actual variability of scrapie prion strains, avoiding confounding effects exerted by strain selection/mutation phenomena in wild-type mice^20,26,38–40,54^.

In our experiment, sheep brains showed a remarkable homogeneity in banding patterns, all of them possessing non-glycosylated (NG) bands with molecular weights of around 19 kDa and a predominance of the diglycosylated (DG) species. This biochemical signature has been linked to the CH1641 isolates, but also to other unconventional isolates found in the field^47–49^. On the other hand, PrP^res^ in isolates prepared from mesenteric lymph nodes reproduced the same pattern in almost all cases; the only exception was isolate 7 L, whose NG band had a molecular weight of 21 kDa.

Concerning transmission properties, most isolates showed survival periods always shorter than 330 dpi on second passage. In contrast, a number of inocula (2 L, 4 L, 5 L, 6 L and 8 L) triggered very protracted survival periods (longer than 481 dpi) and absence of evident reductions on second passage in the Tg388 line. Regardless of whether this difference was caused by reduced accumulation of infectivity in spinal cord of first-passage mice or because they have intrinsically longer survival periods, it suggests the existence of a different strain in this group of samples.

Regarding banding patterns, non-equivalent results were found between TgShp XI and Tg338 lines. TgShp XI mice accumulated high (21-kDa) PrP^res^ in spinal cord after the inoculation of isolates 3 N, 4 N, 5 N and 7 L, although only one of these (inoculum 7 L) displayed this high-molecular weight banding pattern. The prion variant propagated in these cases has been termed “21K-TgShp XI” and did not correlate with other differential phenotypic features such as lesion profiles, vacuolization scores, or PrP^Sc^ distribution patterns.

Another group of isolates (2 L, 4 L, 5 L, 6 L and 8 L) triggered no accumulation or accumulation of a 21-kDa isoform exclusively in Tg338 mice. Notably, these same isolates caused long survival periods, milder spongiosis, flatter lesion profiles, characteristic immunostaining of lateral amygdaloid nuclei and, in a number of cases, presence of PrP amyloid plaques in Tg338 mice. The combination of these differential features allows proposing that a distinct prion conformer, here referred to as “21K-Tg338”, has been propagated in these cases. Upon comparison with other studies, the behaviour of this prion variant resembled that of the scrapie source that caused several outbreaks in Italy in the 90 s following an iatrogenic infection with a vaccine against *Mycoplasma agalactiae*^55–58^, including the accumulation of PrP plaques with a distribution similar to what we observed^58^. In other study, an isolate coded MF17, whose properties resemble those of Italian scrapie (O. Andréoletti, unpublished observations), demonstrated zoonotic potential as it was able to infect transgenic mice expressing human PrP^C43^. Studies are underway to determine if the coincidence of phenotypic traits is enough to endorse our inocula with zoonotic capability.

In the rest of cases, a 19-kDa conformer, likely corresponding to the one predominating in most of the original sheep isolates, was propagated and termed “19K”. Importantly, both murine lines were able to propagate this conformer. The phenotype associated to this form of the agent resembled that of CH1641-like isolates^46,47,59,60^. CH1641 scrapie is associated with a PrP^res^ banding pattern with low (19–20 kDa) NG species and possesses distinctive biological properties. In natural cases, it causes longer incubation periods in sheep with the s7 allele of the *Sip* (*Prnp*) gene and shorter in sheep with the p7 allele, contrary to the more common scrapie sources studied, including SSBP/1^28^. Contrary to other classical scrapie sources, the original, prototypic CH1641 source does not transmit to wild type mice^28^. However, it does transmit without apparent difficulties to transgenic mice expressing ovine PrP^C^, which replicate the PrP^res^ banding pattern of the source inocula^47^, in agreement with our own results. Due to its biochemical features, these isolates were suspected to contain BSE infectivity^46^, although this was later discarded. To date, this type of scrapie has not been attributed a zoonotic potential^49^ (O. Andréoletti, unpublished observations).

All studied isolates seemed to contain the “19K” component, since in all cases at least one of the transgenic lines reproduced the phenotype associated to this isoform. However, TgShp XI propagated the “21K-TgShp XI” component when a number of brain-derived isolates and one lymph node-derived isolate were used. The fact that not all isolates induced the propagation of this variant indicates that the “21K-TgShp XI” component pre-existed in these inocula and was not generated *de novo*. The reason why Tg338 mice were unresponsive to the presence of “21K-TgShp XI” prions and manifested solely the phenotype associated to the major “19 K” component remains unresolved. The possibilities are that it is caused by the PrP^C^ amino acid mismatch between sheep (ARQ PrP^C^) and Tg338 mice (VRQ PrP^C^) or by differences in PrP^C^ expression levels, as recently proposed^61^, and discussed below.

In contrast, half of the lymph node-derived inocula provoked in Tg338 mice a characteristic phenotype that was associated with the propagation of the “21K-Tg338” isoform, which probably pre-existed in these inocula as a minor component. This conformer is somehow able to intercept the propagation of the major “19K” component exclusively in Tg338 mice, strongly protracting survival periods and triggering less severe neuropathology. This interception may be mediated by the formation of PrP amyloid plaques, as observed in previous studies with the Italian isolate^58^ and in this study for inocula 6 L and 8 L. According to some authors, amyloidogenesis may comprise a defensive system by means of which toxic small PrP oligomers^25,34,62^ are sequestered into bigger structures, thus delaying or restraining the neurotoxicity caused by prion progression^63–65^.

Although the conclusion is that there is an intrinsic variability of prion agents in natural scrapie cases that can be resolved by means of bioassay, the mechanisms though which each transgenic murine line selectively propagates distinct conformers are far from clear. In this line, a recent study has described how high-level expression of ovine PrP^C^ favours the selection of minor components in a mixture of prion quasi-species, and moreover, triggers the *de novo* generation of new, favoured conformers^61^. Whether such a mechanism may participate in the divergence observed in our study needs to be evaluated. A possibility is that the ability of each transgenic line to amplify distinct prion conformers is due to their different rates of PrP^C^ expression (4–8-fold *vs*. 8-fold), rather than, or in combination with, differences in the sequence of the PrP^C^ they bear.

Interestingly, the “21K-Tg338” component was only found in lymphoid tissues from sheep. It is widely accepted that, upon natural, oral infection in sheep, prions invade the gut-associated lymphoid tissue and progressively extend to the rest of the lymphoreticular system (LRS)^66,67^. During this process, the LRS may favour the propagation of specific prion conformers, different from the isoforms that later invade the nervous system. This phenomenon has been observed in ovinised mice whose brain and spleen accumulated PrP^Sc^ with differential banding patterns and biological properties^68^. This situation is reminiscent of the case of humanised transgenic mice inoculated with variant CJD (vCJD) prions that developed a disease phenotype compatible with sporadic CJD (sCJD), but still accumulated vCJD PrP^Sc^ in their spleens^69^.

Nevertheless, the reasons why brain and lymphoid organs propagate distinct prion conformers have not been clarified. It has been proposed that differences in the glycosylation of the PrP^C^ between these two tissues may be involved in the selection of PrP^Sc^ isoforms with specific glycoform ratios^70^. In addition, the aforementioned study by Le Dur *et al*. suggests an alternative mechanism based on differences in PrP^C^ expression level^61^.

In any case, our results indicate that in natural scrapie cases, sheep can be infected with more than one strain. These distinct variants of the agent can be present in different organs (we assessed nervous and lymphoid tissues), or even coexist in the nervous system as prion mixtures with a dominant conformer and one or more subdominant isoforms, which can be detected by bioassay in sensitive rodent models or other *in vitro* techniques. In fact, according to our results, the coinfection seems to be more frequent that the infection with a single strain.

The notion that natural scrapie cases may be caused by mixtures of substrains is not new. There are many precedents in the literature: (i) when a methodology that separate short and long-incubation period strains was employed, natural scrapie isolates were found to contain a mixture of strains that could be isolated in different lines of wild type mice^54^. (ii) When several wild-type and transgenic lines were used in a bioassay to investigate a number of scrapie isolates suspected to contain BSE infectivity, different results with lines TgShp XI and Tg338 were obtained, which suggest that different strains existed in the isolates that each mouse line propagated selectively^71^. (iii) When CH1641-like scrapie isolates were inoculated in wild-type mice, transmission was achieved in disagreement with previous reports, although glycosylation profiles and histopathological features changed. These results were compatible with a selection of a minor component that was able to replicate in wild-type mice, in contrast with the CH1641 experimental isolate^72^. (iv) Other authors^73^ also worked with CH1641-like isolates and found them to be comprised of an ensemble of different conformers than could be differentiated by the use of Western blot and separately propagated in distinct sheep breeds and murine lines. All these results are in agreement with ours and drag the risk of sheep getting infected with multiple scrapie strains into the spotlight.

This situation is somehow similar to some cases of sporadic CJD (sCJD) in humans, which were shown to possess a mixture of high and low-molecular weight conformers in their brains or in different parts of their brains^74^. Even in variant CJD (vCJD) cases, 21-kDa PrP^Sc^ conformers have been occasionally found^75^, which raises doubts about the current classification of human prion strains and the actual responsible of each type of clinical profile.

Characterization and monitoring of prion strains in small ruminant populations in Europe is crucial for control and eradication purposes. Shedding light on the actual variability of scrapie prions and its implications for inter-species transmission and, particularly, on the zoonotic potential of scrapie field isolates is necessary to update communitarian public health policies and to prevent a putative reemergence of prion diseases as a public hazard.

## Methods

### Sheep

The diagnosis of scrapie in terminally-affected sheep was stablished using standardized clinical examination. In contrast, preclinical and early clinical animals were identified *in vivo* thought rectal biopsy and immunohistochemical detection of PrP^Sc^ on rectal mucosa-associated lymphoid tissue using monoclonal anti-PrP antibody L42 (R-Biopharm), as described elsewhere^76^.

Animals were sacrificed by intravenous injection of sodium pentobarbital followed by necropsy and systematic sampling. Samples from brain and mesenteric lymph nodes were divided into two halves; one half was fixed in a solution of 10% formalin for further histological studies, while the other was immediately immerged in liquid nitrogen and later conserved at −80 °C for biochemical analyses and the preparation of inocula.

After sacrifice, the presence of PrP^Sc^ was confirmed for both the terminal and the preclinical/early clinical group in both nervous and lymphoid tissues using immunohistochemistry with monoclonal anti-PrP antibody L42 (R-Biopharm), whose epitope spans amino acids 145–163 of ovine PrP. Additionally, the *Prnp* genotype was determined through sequencing of genomic DNA obtained from whole blood samples.

### Inocula

First-passage inocula were prepared from nervous (N) and lymphoid tissues (L) of the aforementioned sheep. Inocula 1 N to 10 N corresponded to inocula prepared from medulla oblongata (obex), while inocula 1 L to 10 L were those prepared from mesenteric lymph nodes of the animals. Inocula 1 N to 6 N and 1 L to 6 L derived from tissues of terminal sheep, while inocula 7 N to 10 N and 7 L to 10 L sourced from preclinical / early clinical sheep. Second-passage inocula were prepared from pools of spinal cords harvested from diseased first-passage mice. All inocula consisted of 10% (w/v) tissue homogenates in physiological saline and were subjected to microbiological analysis to ensure sterility prior to intracerebral inoculation.

### Mice

TgShp XI mice express ovine ARQ PrP^C^ with expression levels of between 4 and 8-fold compared to that of sheep brain^41^. These animals were kindly provided by M. Groschup (Friedrich-Loeffler-Institut, Greifswald - Insel Riems, Germany) and were brought to our facilities to be inoculated after the appropriate adaptation period.

The transgenic murine line Tg338 was developed by H. Laude and J.L. Vilotte (INRA, Jouy-en-Josas, France)^42^ and is homozygous for the ovine *Prnp* gene, VRQ allele, expressing ovine VRQ PrP^C^ under the control of the ovine PrP promoter^37^. The level of expression of this line is approximately 8-fold that of sheep brain. This line was provided by O. Andréoletti (ENVT-INRA, Toulouse, France) and was maintained and bred in our facilities.

All experimental procedures in this study were approved by the Ethics Committee for Animal Testing of the University of Zaragoza (permit number PI19/14) and performed in accordance with the recommendations for the care and use of experimental animals and in agreement with Spanish law (RD 1201/05).

### Intracerebral inoculation

A dose of 20 µl/animal of each inoculum was administered by the intracerebral route to groups of six mice using a precision syringe and under general anaesthesia. Animals were provided adequate analgesia after the procedure, consisting of buprenorphine at a dose of 0.01 mg/kg bodyweight by the subcutaneous route, and were caged together.

### Bioassay

Animals were monitored three times per week for clinical signs of prion disease. When the end point criteria were met, animals were sacrificed by cervical dislocation under heavy anaesthesia. Brain and spinal cord were harvested and stored in a 10% formalin solution and at −80 °C for histopathological and biochemical analyses, respectively.

### Western blotting

Western blotting was performed following a protocol based on TeSeE Western Blot kit (Bio-Rad). Briefly, medulla oblongata and mesenteric lymph node samples from sheep and spinal cord pools from diseased mice were thawed and homogenised in a detergent-containing solution. A volume of 200 µl was submitted to proteinase K digestion for 10 min, which was stopped using a β-mercaptoethanol-containing stop buffer, followed by concentration, clarification and resuspension of the remaining PrP^res^ in 30 µl of Laemli loading buffer. It was then subjected to SDS-PAGE electrophoresis using commercial 12% Bis-Tris gels (Bio-Rad), followed by transference to a PVDF membrane with 0.20-µm pore diameter (Bio-Rad). Immunoblot was performed by sequentially immerging the membrane in a blocking solution (0.2% BSA in PBS+Tween 0.1%), primary anti-PrP antibody Sha31 (whose epitope spans amino acids 148–155) diluted 1:8,000 in PBS+Tween 0.1%, and HRP-conjugated secondary antibody diluted 1:5,000 in PBS+Tween 0.1%. Finally, membranes were developed by incubation with a luminol-based substrate (SuperSignal West Pico Chemiluminescent Substrate).

### Tissue processing

Brain and lymph node samples from sheep and brains from diseased mice were fixed in a 10% formalin solution for at least 48 hours before being processed. Mouse brains were trimmed in four sections following Fraser and Dickinson’s protocol^15^, while sheep tissues were processed as usual^18^. Tissues were then embedded in paraffin wax and mounted in histological cassettes. Four µm-thick sections were obtained using a microtome and mounted on glass slides for subsequent histological procedures.

### Haematoxylin and eosin staining

Haematoxylin and eosin (H&E) staining of the sections was performed following a standard protocol. Briefly, dewaxing and rehydration was performed by sequentially passaging the preparations in xylene and graded alcohols, followed by incubation in a haematoxylin solution. After rinsing with tap water, preparations were subjected to incubation in acid alcohol (1% acetic acid in a 70% ethanol solution), followed by immersion in an eosin solution. Finally, preparations were dehydrated and mounted prior to visualization under light microscope.

### Immunohistochemistry

Immunohistochemistry was applied to brain and lymph node samples from sheep and brain sections from mice. After dewaxing and rehydration, three different pre-treatments for antigen retrieval were performed sequentially: immersion in 98% formic acid for 15 min, treatment with 4 µg/ml proteinase K for 15 min at 37 °C, and hydrated autoclaving in citrate buffer at 96 °C for 20 min. Following antigen retrieval, endogen peroxidase activity was blocked using a commercial blocking solution, followed by 1-hour incubation with primary anti-PrP antibody L42 (1:500, R.Biopharm) for sheep tissues or 6H4 (epitope aa 147–155, 1:100, Prionics) and SAF84 (epitope aa 160–170, 1:1,000, SPI-Bio) for mice brains. The EnVision+ System (Agilent Dako) was used as the secondary antibody, and the DAB + System (Agilent Dako), based on the use of 3,3’-diaminobenzidine (DAB) as chromogen, was employed for development.

### Paraffin-embedded tissue-blot (PET-blot)

PET-blot was performed as described elsewhere^77^ on mice brain samples. Briefly, 4-µm paraffin-embedded brain sections were collected onto a nitrocellulose membrane and dried at 37 °C for 24 hours. Membranes were then subjected to dewaxing and rehydration and incubated for 2 hours in a solution of proteinase K (250 µg/ml) at 56 °C to completely digest PrP^C^. Denaturation of the remaining PrP^res^ was achieved by incubating the membranes in a solution of guanidine thiocyanate 3 M. After blocking the membrane with 0.2% BSA to avoid cross-reactivity, detection was carried out through sequential incubation with the anti-PrP antibody Sha31 (1:8,000, SPI-Bio) and a secondary alkaline phosphatase (AP)-conjugated antibody (1:500, Agilent Dako), followed by development with NBT/BCIP (Thermo Scientific). Membranes were then washed and dried for 24 hours at room temperature.

### Lesion and PrP^Sc^ distribution profiling

H&E-stained and PET-blotted brain samples from second-passage animals were inspected and spongiosis and PrP^Sc^ accumulation were was measured in nine pre-determined grey matter areas following a standardized protocol^15^. The areas were: (1) dorsal nuclei of medulla oblongata, (2) cerebellar cortex, (3) superior colliculus of the mesencephalon, (4) hypothalamus, (5) thalamus, (6) hippocampus, (7) lateral septal nuclei, (8) cerebral cortex at the level of the thalamus and (9) frontal cortex. Semiquantitative scores from 0 (absence of vacuolization or PrP^Sc^ accumulation) to 5 (very abundant and confluent vacuoles or PrP^Sc^ deposits) were given to each area. Mean values at each area were plotted to trace the curves for each inoculum. Visual comparison was performed between curves corresponding to each inocula, as well as between lesion profiles and or PrP^Sc^ distribution curves. In addition, the statistical correlation between both parameters was computed through the Spearman’s correlation coefficient for each transgenic line. The software used was GraphPad Prism 5.

## Supplementary information

Supplementary information.

Supplementary information.
