# Supplementary material for: Mixtures of prion substrains in natural scrapie cases revealed by ovinised murine models
Source: Sci Rep. 2020 Mar 19;10:5042. doi: 10.1038/s41598-020-61977-1 (PMC7081250; doi:10.1038/s41598-020-61977-1)

## Supplementary information

# Mixtures of prion substrains in natural scrapie cases revealed by ovinised murine models

Tomás Barrio[1], Hicham Filali[1], Alicia Otero[1], Jessica Sheleby-Elías[1], Belén Marín[1], Enric Vidal[2], Vincent Béringue[3], Juan María Torres[4], Martin Groschup[5], Olivier Andréoletti[6], Juan José Badiola[1], Rosa Bolea[1,*]

[1] Centro de Encefalopatías y Enfermedades Transmisibles Emergentes, Facultad de Veterinaria, Instituto Agroalimentario de Aragón - IA2 (Universidad de Zaragoza - CITA), 50013 Zaragoza, Spain

[2] Priocat Laboratory, Centre de Recerca en Sanitat Animal (CReSA), UAB-IRTA, Universitat Autònoma de Barcelona (UAB), 08193 Bellaterra, Barcelona, Spain

[3] UMR Virologie Immunologie Moléculaires (VIM-UR892), INRA, Université Paris-Saclay, 78352 Jouy-en-Josas, France.

[4] Centro de Investigación en Sanidad Animal, CISA-INIA, 28130 Valdeolmos, Madrid, Spain.

[5] Institute of Novel and Emerging Infectious Diseases, Friedrich-Loeffler-Institute, Südufer 10, 17493 Greifswald-Isle of Riems, Germany.

[6] UMR INRA ENVT 1225- IHAP, École Nationale Vétérinaire de Toulouse, 31076 Toulouse, France.

**Supplementary Figure S1**

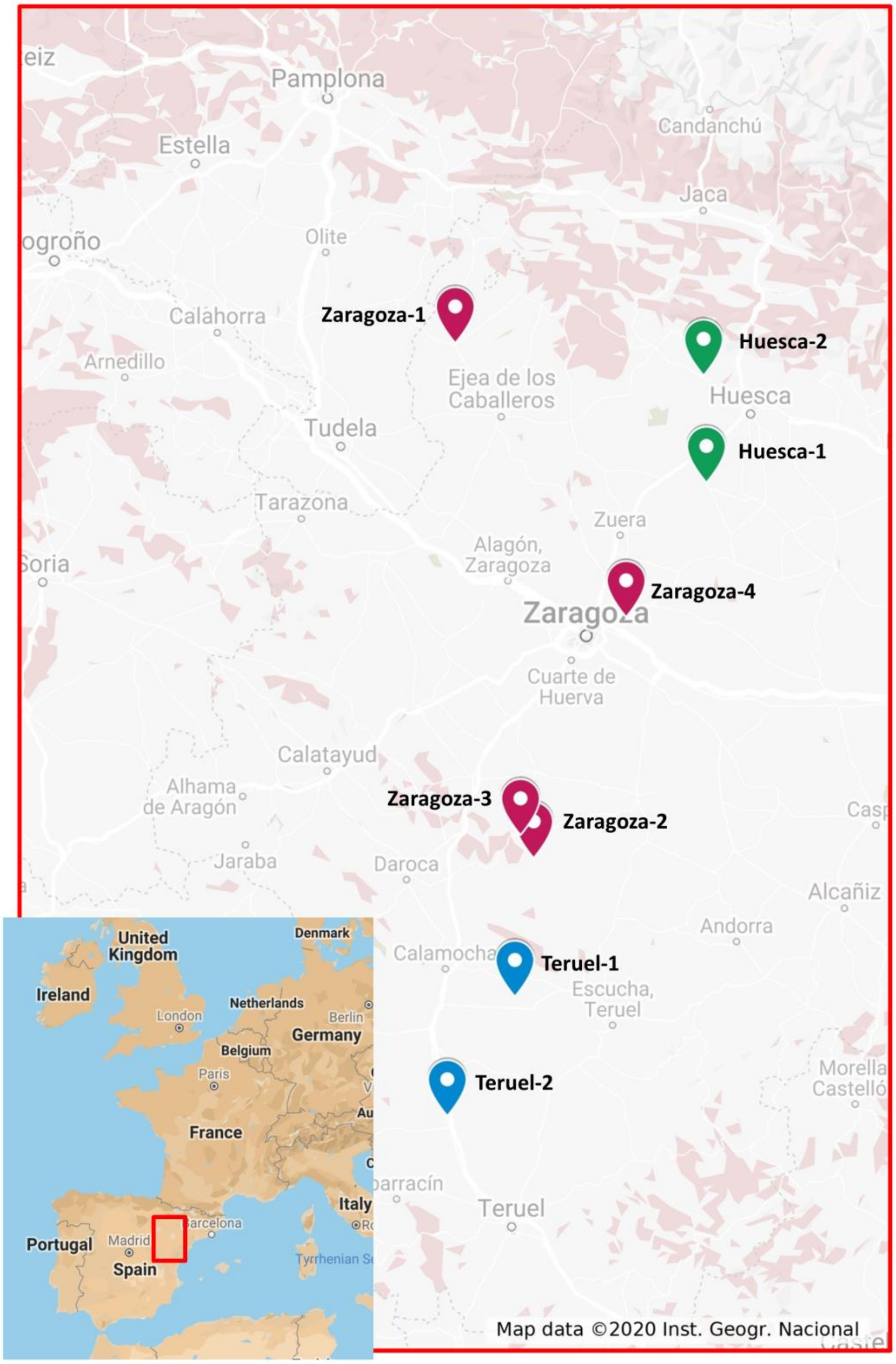

**Supplementary Figure S2**

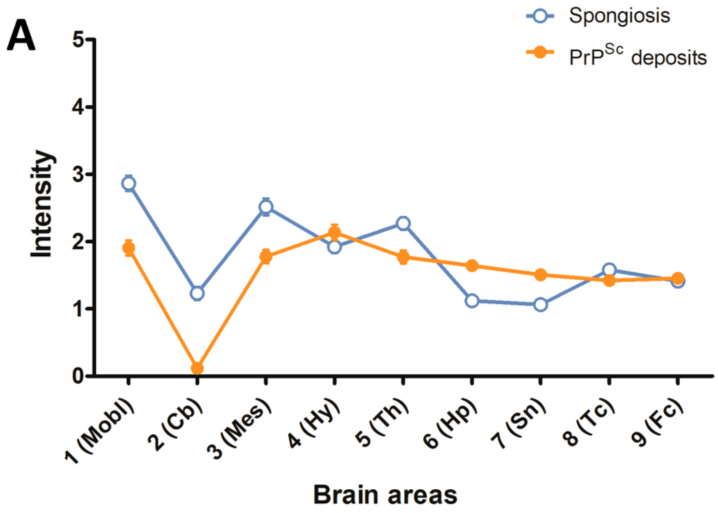


r = 0.5651 ; $p < 0.0001$

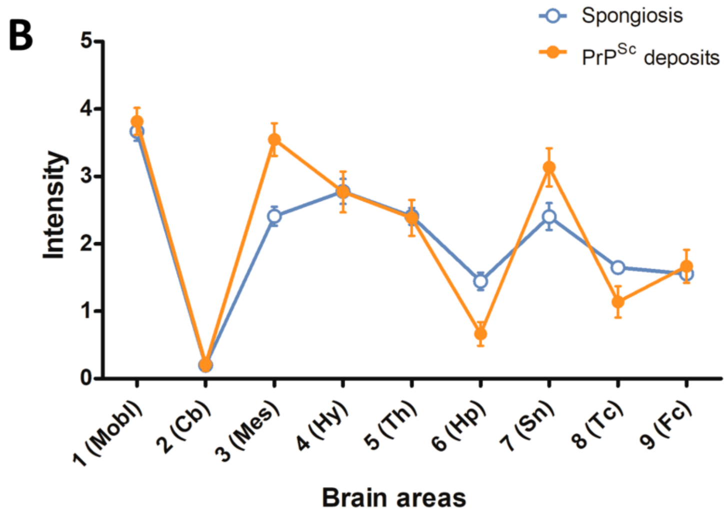


r = 0.3245; $p < 0.0001$

**Supplementary Figure S3**

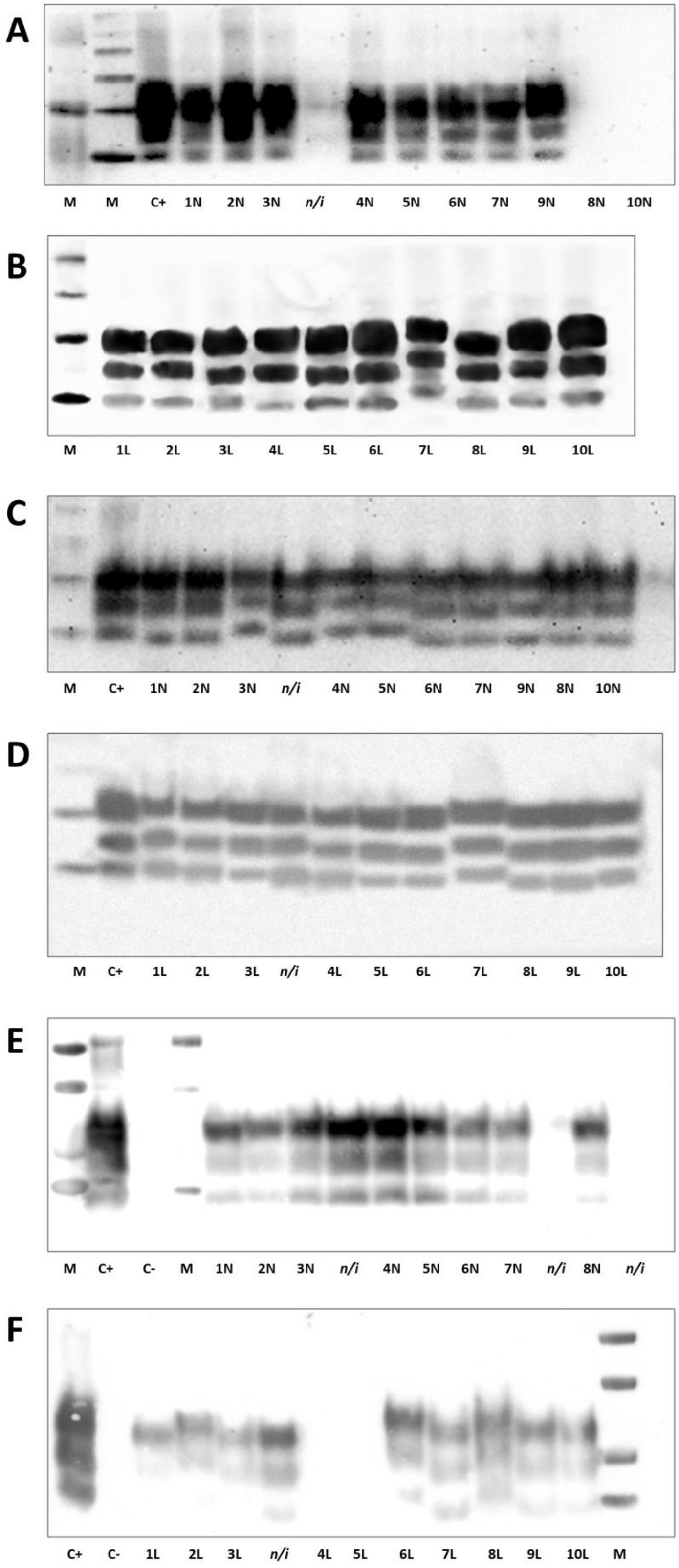

Supplement: Supplementary file 1 — Supplementary information. [file 41598_2020_61977_MOESM1_ESM.pdf]
