# Supplementary material for: Mixtures of prion substrains in natural scrapie cases revealed by ovinised murine models
Source: Sci Rep. 2020 Mar 19;10:5042. doi: 10.1038/s41598-020-61977-1 (PMC7081250; doi:10.1038/s41598-020-61977-1)
Supplement: Supplementary file 2 — Supplementary information. [file 41598_2020_61977_MOESM2_ESM.docx]

**Supplementary Figure S1.** Map of the Aragón region within the Spain-France-Andorra transboundary territory showing the geographical location of the flocks wherefrom the scrapie-infected sheep were obtained. The map was obtained from Google Maps and the data provider appears in the lower part of the image.

**Supplementary Figure S2.** Distribution of spongiform lesions and PrP^Sc^ deposits in the brain of TgShp XI and Tg338 mice and correlation analysis between them. Curves were plotted from the average vacuolization or PrP immunostaining intensity for each transgenic line. At least three animals for each challenged group were included. The correlation analysis was performed using the non-parametric Spearman’s correlation coefficient (r). Mobl: medulla oblongata, Cb: cerebellar cortex, Mes: mesencephalon, Hy: hypothalamus, Th: thalamus, Hp: hippocampus, Sn: septal nuclei, Tc: cortex at the level of thalamus, Fc: frontal cortex. Error bars represent SEM.

**Supplementary Figure S3**. Original, full images of Western blots from Figures 1, 2 and 3. *M*: molecular weight marker, *C+*: positive control, *C-*: negative control, *n/i*: not included (lane not included in the study).
